# Evaluating the effective segmentation of human lateral geniculate nucleus

**DOI:** 10.1007/s00429-025-03000-9

**Published:** 2025-08-25

**Authors:** Lauren E. Welbourne, Joel T. Martin, Federico G. Segala, Anisa Y. Morsi, Daniel H. Baker, Alex R. Wade

**Affiliations:** 1 Department of Psychology, University of York, Heslington, York, UK; 2 School of Philosophy, Psychology and Language Sciences, University of Edinburgh, Edinburgh, UK; 3 York Biomedical Research Institute, University of York, Heslington, UK

**Keywords:** LGN, Thalamus, Segmentation, MRI, Proton density, Vision

## Abstract

Important parts of the visual pathway occur in relatively small subcortical structures that are often difficult to identify and segment using standard structural scans in MRI (e.g. T1 and T2 scans). Studies of the Lateral Geniculate Nucleus (LGN) often use proton density (PD) scan protocols, repeated up to 40 times, then manually segment the LGN structure from the average image. Efficiency is crucial when conducting MRI scans: minimising time spent on structural scanning can increase time available for complementary functional MRI scans and/or reduce scanning costs. In this study we asked how segmentation accuracy depended on the number of PD repeats. Four raters segmented the LGN of five participants, using different numbers of PD scans in the average image (1, 2, 4, 8, 16, 24, 32, 40), and an additional experienced expert rater segmented the LGN for just the 40PD average for all participants. We compared how the rater LGN masks at each scan average level overlapped with the expert masks. One rater performed the segmentation for the 40PD average on four separate days, to measure intra-rater variability across repeats. We also used a state-of-the-art automated segmentation process to compare the reliability to manual segmentation. We found that the average overlap between rater masks and the expert masks increased up to the 16PD scan average level, after which there was no additional benefit to including more PD scans. The automated segmentation masks were comparable to the overlap between the raters (40PD) and expert masks.

## Introduction

The lateral geniculate nucleus (LGN) is a pre-cortical thalamic structure that relays information from the retina to the visual cortex, collates information from the left and right eyes, and also receives input from the cortex via feedback connections (Jones 1985; Sherman and Guillery 2001). It is often of interest to compare the relative size of the LGN between individuals with and without particular congenital conditions (e.g. albinism (Grigorian et al. 2016; Mcketton et al. 2014)) or degenerative conditions (e.g. Alzheimer’s disease (Erskine et al. 2016; Forno et al. 2023; Iglesias et al. 2018)). Furthermore, functional imaging studies measure differences in functional activity within this area in response to different stimuli, to understand what information is processed within the LGN (Chai et al. 2019; Mullen et al. 2010; Yu et al. 2016).

Structural scans obtained during MRI/fMRI experiments typically use T1 and T2-weighted contrasts to produce a high-quality full brain image, which are reconstructed in pre-processing stages to give a detailed 3D brain image, onto which functional data can be mapped (Dale et al. 1999; Smith et al. 2004). However, these scans are not optimised for delineating subcortical structures like the LGN. Therefore, proton density (PD) scans are often used to enhance the contrast in the thalamus and make it possible to segment the LGN structure (Devlin et al. 2006; Fellner et al. 1994; Schumann et al. 2001). To further improve the contrast of the image (by increasing the signal to noise ratio (SNR)), it is common practice to obtain multiple PD images - sometimes > 40 - which are aligned and then averaged together to produce a clearer visual of the LGN in the scan image (DeSimone and Schneider 2019; Giraldo-Chica and Schneider 2018; Mcketton et al. 2014; Oishi et al. 2023). Increasing the number of PD scans in an average image necessarily reduces the image noise (with diminishing return as scan numbers get higher) (Mcketton et al. 2015), but it has not been demonstrated that the improvement in SNR continues to improve the resulting LGN segmentation.

It takes a minimum of 1 h of scan time to collect 40 PD images, of the type often used for visualising the LGN (a set of coronal slices which covers approximately 5 cm over the region containing the LGN). In experiments where it is also of interest to collect functional data, this is a significant amount of additional scan time, which increases both the time cost to experimenters and participants, as well as the monetary cost of scanning time. To optimise these costs, it is prudent to examine the minimum number of scans required to achieve the same end result of a clearly segmented LGN. It should also be considered whether manual segmentation from numerous PD scans are required at all, as modern auto-segmentation toolboxes allow the subcortical thalamic areas to be segmented using just the structural T1 images.

Here we investigate segmentation accuracy as a function of the number of PD scans included in the average image. We perform an inter-rater and intra-rater analysis, using an expert rater as our ‘ground truth’ LGN segmentation in each participant, as well as a comparison to LGN segmentations produced via automated segmentations. Using the Dice (1945) coefficient as a measure of the overlap between rater segmentations and the expert segmentations, we found that there was no further improvement in Dice score after the 16PD scan average level, indicating that 16 PD scans would produce as reliable an LGN segmentation as 40 PD scans. There were significant differences between the intra-rater segmentation repetitions, with an increase in Dice score between the first and subsequent repetitions, indicating an improvement in LGN segmentations with repeated exposure. Automated segmentations were compared to the expert segmentations using the same Dice coefficient method, and produced average Dice scores that were almost identical to the rater vs. expert Dice score for the 40PD scan average level.

## Methods

### Participants

Five participants (2 female, age = 38.4 ± 8.2 years) took part in this experiment. Each participant performed a single structural scanning session, lasting approximately 1.5 h. For their entertainment and comfort during the session, the participants watched videos of their choice (with subtitles) during the scans, via a head-mounted mirror and projector. The study protocols were approved by the Research Governance Committee of the York Neuroimaging Centre at the University of York (Project P1486).

### MRI protocols

A 3-Tesla Siemens Magnetom Prisma MRI scanner and a Siemens 32-channel head and neck coil were used to acquire all scans, at the York Neuroimaging Centre (YNiC, University of York, UK). High resolution structural scans were acquired at the start of the session: one T1-weighted (TR 2400ms, TE 2.32ms, voxel size 0.75 × 0.75 × 0.75 mm, 8° flip angle, FOV 240 × 240 × 144 mm, 192 slices), and one T2-weighted (TR 3200ms, TE 562ms, voxel size 0.75 × 0.75 × 0.75 mm, FOV 240 × 240 × 156 mm, 208 slices, aligned from the centre of the slice group from the T1-weighted scan prescription). These were followed by 40 coronal proton density (PD) scans, acquired using the same protocol used in Oishi et al. (2023) (TR 3000ms, TE 21ms, 120° flip angle, FOV 192 × 192 mm, matrix size 256 × 256, 49 coronal slices, slice thickness 1 mm (no gap), voxel size 0.75 × 0.75 × 1 mm, in-plane acceleration factor of 2) – PD scans were run in four batches of 10 (approximately 15 min per batch), with the MRI operator communicating with the participant after each batch.

### MRI data processing

The T1 and T2 structural images were used in the 3D reconstruction of the brain, which was performed with Freesurfer’s (http://surfer.nmr.mgh.harvard.edu/) (Dale et al. 1999; Reuter et al. 2012) *recon-all* command, using the “-hires” flag (used for sub-millimeter voxels), which required an additional “-expert” flag to input an expert.opts file, specifying the maximum number of iterations (50) to perform during the inflation of the cortical surface (“mris_inflate -n 50”).

The PD scans were all aligned to the first PD scan of the session (the ‘reference’), using FSL’s (http://fsl.fmrib.ox.ac.uk/fsl/fslwiki/*)* (Smith et al. 2004) *flirt* command to perform a rigid-body transformation. Average PD scans were then created for each of our PD scan levels, where different numbers of PD scans were used in the average, using *fslmaths* (an FSL program). The scan levels used were: 1, 2, 4, 8, 16, 24, 32, and 40. These scan levels, which are the number of scans in the average, will be referred to by their number followed by ‘PD’, for example, 40PD used all 40 PD scans in the average. When creating the averages, the necessary number of scans were taken from the scan list in chronological order, i.e. for 8PD, the first 8 scans from the session were averaged.

### Raters and manual segmentation procedure

**Fig. 1:**
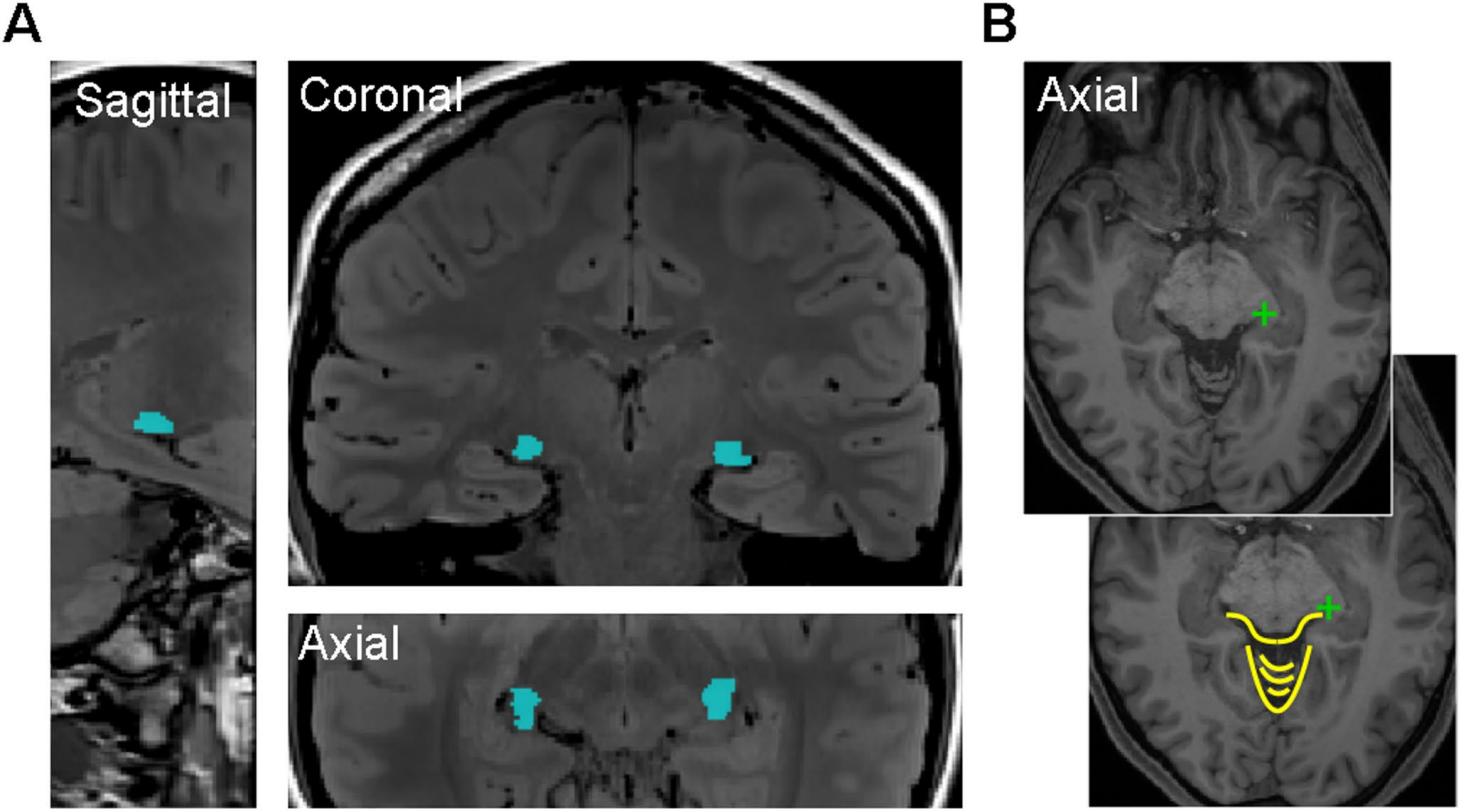
Example of LGN location for one participant from this experiment (**A**), presented in sagittal, coronal, and axial views, from the average of 40 PD-weighted scans. LGN locations are highlighted by the overlaid blue mask. **B** Axial slice from the T1 scan demonstrating the ‘bee’ used for navigating to the location of the LGN - both images are the same, with a green cross marking the point used to navigate to the left (radiological view) LGN, and the bottom image showing the outline of the ‘bee’ body and antennae in yellow. After navigating to this point in the axial view, the coronal view would then be used to identify and draw the LGN segmentation for each coronal slice

The four raters (all authors), were experienced in neuroimaging analysis and associated visualisation/masking tools, but none had prior experience in LGN segmentation. The raters performed a group training session together, using previous papers that included clear examples of the LGN location (DeSimone et al. 2015; DeSimone and Schneider 2019; Fujita et al. 2001; Kastner et al. 2004; Oishi et al. 2023) to first learn how to navigate to the likely location of the LGN (moving toward the top of the cerebellum in the axial view, which looks like the body of a striped bee, and following to the end of each line of cerebrospinal fluid (CSF) sitting anterior to the top of the cerebellum, which look like the antennae of the ‘bee’, see Fig. 1B), and to then identify the characteristic ‘comma’ shape (the narrow end pointing laterally in each hemisphere) of the LGN in the coronal view (Fig. 1A). During this training session all raters became familiar with the location and appearance of the LGN, and were all confident on the anatomical landmarks used to navigate to the LGN location. The raters then performed the segmentations for each PD scan level independently, without conferring. A randomised participant order was allocated to each rater - both hemisphere LGN segmentations for each PD scan level were completed in order, starting with using just the T1 image on its own (the ‘0PD’ level) and then working through the PD scan average levels (i.e. 1PD to 40PD) before moving on to the next participant. Once a level was completed, the raters were not permitted to go back and edit their segmentation masks after seeing subsequent PD average levels (to avoid bias).

After completing all segmentations, one of the raters then repeated the 40PD LGN segmentations on three more occasions (different days) for each participant (total of four repeats per participant), as a measure of intra-rater reliability.

An expert rater, an author of a recent publication focused on the LGN, with experience in segmenting the LGN, also performed segmentations for each hemisphere in each participant, for the 40PD level. These segmentations were used as our comparison/’ground truth’ LGN masks, to compare to all other segmentations (raters and automated). The expert rater performed each segmentation once for each participant, and therefore we do not have a measure of intra-rater reliability for the expert rater.

### Automated segmentation

We compared our manual segmentations against a modern automatic segmentation algorithm (‘HIPS-THOMAS’ (Vidal et al. 2024)) that has previously shown high accuracy and specificity. The algorithm, which we ran within a docker container for maximum reproducibility, is available at https://github.com/thalamicseg/hipsthomasdocker. Implementation details are available in the repository and in the accompanying paper. We ran the HIPS-THOMAS algorithm on the single, unaveraged pre-processed Freesurfer T1 images (‘T1.mgz’) for each participant at a resolution of 1 × 1 × 1 mm. For each participant, this algorithm provided a left and right binary LGN mask. We compared the automated masks to those from our manual segmentations as described below. In this algorithm, it is not possible to include PD images for the segmentation, and therefore a direct comparison of how the automated segmentation may perform with the same scans used by the human raters is not possible here.

### Data analysis

The volume of the LGN in each hemisphere (across participants and independent raters) was extracted for each PD scan level (0PD to 40PD), and for the expert and automated segmentations, using the *fslstats* tools from the FSL package.

In order to quantify the accuracy of segmentations across PD scan average levels, we used the expert rater’s masks for each participant as the participant’s ‘ground truth’ LGN, and then measured the amount of overlap between each independent rater mask (and each automated segmentation) with the expert rater mask (using *fslmaths* tools to combine the masks, and MATLAB to extract the number of voxels featured in both masks, i.e. those voxels with a value of 2, in the ’overlapRegion’). We used the Dice coefficient as a measure of overlap (the Dice score) (Dice 1945), which is calculated as (2*overlapRegion)/(rater_mask_+expert_mask_), where the overlapRegion is the sum of all voxels that *both* the expert and the rater include in their mask, and the rater_mask_ and expert_mask_ are the total number of voxels in each of the rater and expert masks, respectively.

We used the Kolmogorov-Smirnov test to check for a normal distribution of data points within each PD level (for Dice scores, intra-rater repeats, and LGN volumes) across all participants and raters, and found that the distributions were significantly different from normal. As such, we have plotted medians and the interquartile ranges for the data, and performed Wilcoxon signed rank tests between each PD scan level and the 40PD level, for both LGN Volume and Dice scores, to measure whether the values significantly differed from the maximum number of PD scans in the average (40PD) with increasing PD scans in the average. For all tests, except 40PD vs. expert in the LGN volume tests and 40PD vs. automated segmentation in the Dice Score tests, individual data points (for participants, raters and hemispheres) were included in the test; in the aforementioned exceptions, in order to have matched sample sizes, the median across raters was used for each participant from the 40PD dataset. Significance values were corrected for multiple comparisons using false discovery rate (FDR) correction. One FDR correction was applied to all of the rater tests, and a separate correction was applied to the automated segmentation vs. expert t-tests, and the expert vs. 40PD tests.

## Results

**Fig. 2:**
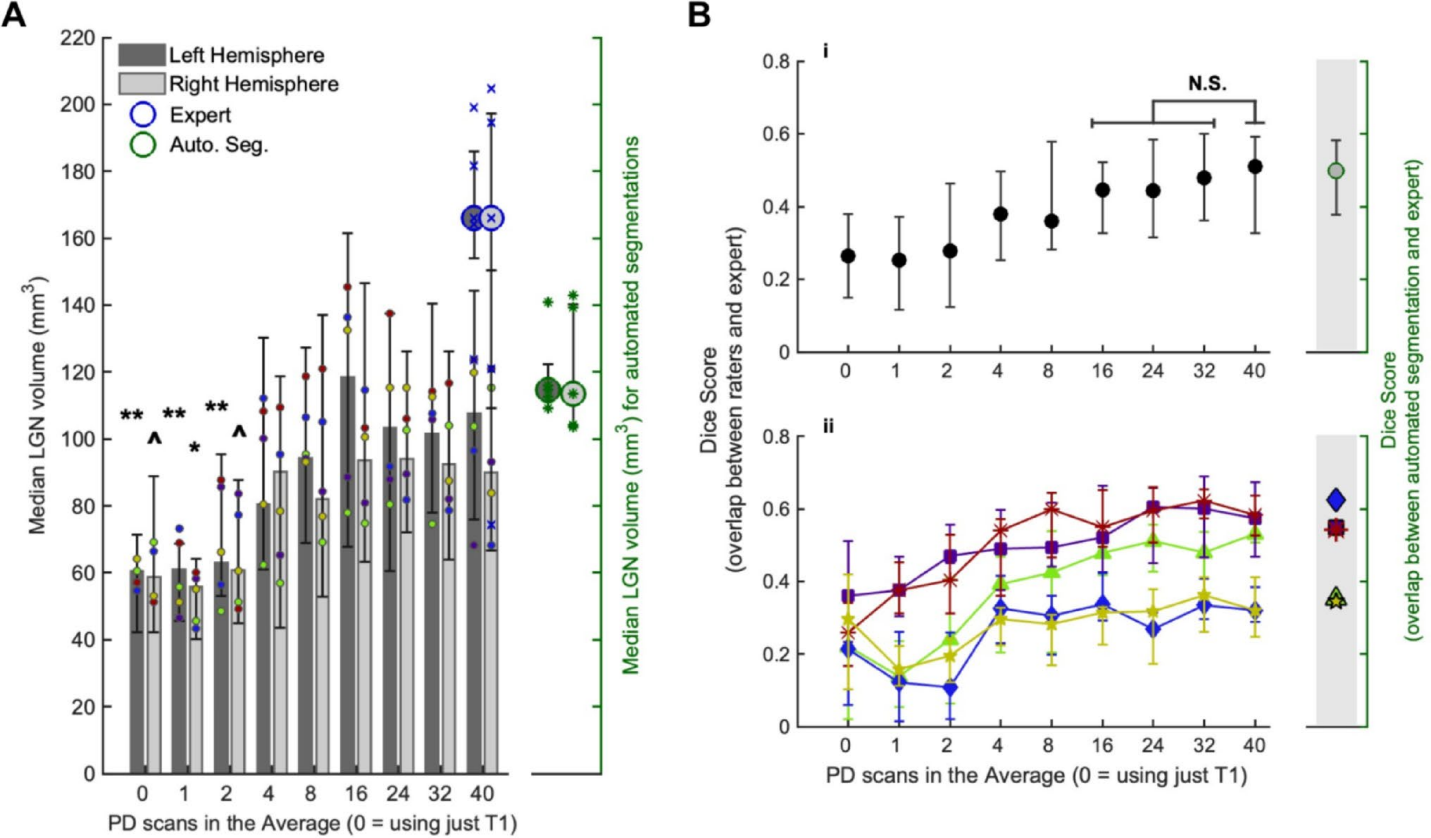
**A** Median LGN Volume across PD scan levels split by hemisphere, across raters and participants, with interquartile range bars. Individual participant medians across raters per hemisphere overlaid in coloured circles (colours match the participant colours used in **B **ii) Median LGN volume across participants from the expert rater are overlaid for the 40PD level in blue circles for each hemisphere, with the individual participant volumes overlaid in blue crosses. The median volumes from the automated segmentations are shown on the right axis in green circles for each hemisphere, with individual participants values overlaid in green asterisks. The left and right axis scales are identical. Significant differences in LGN volume compared to the 40PD scan level are indicated for each hemisphere (^*p* < 0.05, *p < 0.01, **p < 0.001) (Wilcoxon signed rank tests with false discovery rate (FDR) correction for multiple comparisons across hemispheres), all other differences were non-significant (see Table 1). **B** Dice scores, measuring the overlap between rater and expert LGN masks, presented across PD scan levels. The Dice scores for the automated segmentations (vs. expert LGN masks) are shown on the right axis, on the same scale. (i) Median Dice score across participants, with interquartile range bars - Wilcoxon signed rank tests between each level compared to the 40PD level (with FDR correction) were performed, with all levels up to and including 8PD showing a significance level of *p* < 0.05, non-significant pairs are indicated with N.S (see Table 2). (ii) Dice scores per participant (median across raters with interquartile range bars), shown in different colours and symbols for each participant. All plots show data from ‘0PD’, which refers to LGN masks created using just the T1 image and no PD scans (i.e. drawn using only information from landmarks to determine the likely location of the LGN). Automated segmentations were performed using only the T1 image

Across the range of PD scan levels, there was a significant increase in the median LGN volume in each hemisphere, between the 0PD, 1PD and 2PD levels compared to 40PD (Wilcoxon signed rank tests with false discovery rate (FDR) correction across all tests and hemispheres, Table 1), shown in Fig. 2A. There was no significant difference in LGN volume from the 4PD level onwards, compared to 40PD. There was also a trend for the expert rater to draw larger LGN masks, however there was no significant difference between their LGN volumes and the participant volumes across raters for the 40PD level. The average LGN volume in each hemisphere is comparable to those reported elsewhere in the literature (Andrews et al. 1997; Simmen et al. 2022), and we show here that with a single PD scan image, raters are more likely to draw more conservative LGN masks that are smaller than those drawn from images with more PD scans in the average. As a measure of intra-rater consistency, we looked at the correlation between left and right hemisphere volumes across all participants and PD levels (from 1PD to 40PD) for each rater. All raters showed significant correlations (*p* < 0.001), with a range in Kendall Tau between 0.43 and 0.60.

Across all independent raters, the expert rater, and the automated segmentation, for all participants and hemispheres, the location of the LGN aligned closely - all raters correctly identified the locus of the LGN. To determine how the accuracy of the LGN masks changed with increasing PD scans in the average image (the PD scan level), the overlap between the rater LGN masks and the expert rater masks was calculated for each participant, to produce a Dice score for each PD scan level. Figure 2B shows the median Dice score across PD scan levels, with the top panel (i) showing the median across participants, and the bottom panel (ii) showing the same data for each participant (median across raters). The difference between each PD level and the 40PD scan level was determined using Wilcoxon signed rank tests (with FDR correction) (Table 2). All levels up to and including 8PD had significantly lower Dice scores than 40PD. From 16PD onwards, there were no significant differences in the Dice score compared to 40PD, suggesting that there were no further improvements in the accuracy of the LGN masks drawn by our independent raters compared to the expert rater masks after the 16PD level. The 0PD scan level refers to LGN masks drawn using only the T1 image - so the masks were drawn predominantly on knowledge of landmarks and anticipated location of the LGN. Dice scores overlapped strongly between the 0PD, 1PD, and 2PD scan levels, indicating that one or two PD scans do not provide additional clarity on the LGN position beyond what can be estimated using nearby landmarks on the T1 image alone.

**Table 1:** Descriptive statistics (median and interquartile range (IQR)) for the LGN volume (mm^3^) across participants, for each hemisphere (Hemi). The PD level is the number of PD scans in the average, where 0PD means only the T1 structural image was used for the Segmentation. For the Wilcoxon signed rank tests, each PD level, and the expert rater LGN volumes, were paired with 40PD (therefore there are no statistics for 40PD), and the automated Segmentation LGN volumes were paired with the expert masks. For all tests, except the 40PD Vs. expert, individual data points were included in the test (i.e. 5 participants x 4 raters, *N* = 20); for the 40PD Vs. expert test, in order to have matched sample sizes, the median across raters was used for each participant for 40PD in the test

PD level	Hemi	Median volume (mm^3^) (IQR)	*N*	W	z	Sig. (FDR)
0PD	Left	60.47 (42.19–71.44)	20	9	−3.584	**< 0.001**
	Right	58.78 (42.19–88.88)	20	39	−2.464	**0.037**
1PD	Left	61.03 (45.56–68.63)	20	6	−3.696	**< 0.001**
	Right	55.69 (40.22–64.12)	20	8	−3.622	**0.002**
2PD	Left	63.00 (53.16–95.34)	20	9	−3.584	**< 0.001**
	Right	60.75 (45.00–57.75)	20	29.5	−2.819	**0.019**
4PD	Left	80.44 (61.03–130.22)	20	56.5	−1.811	0.140
	Right	90.28 (43.59–118.69)	20	69	−1.344	0.286
8PD	Left	94.22 (68.91–127.41)	20	83	−0.821	0.470
	Right	82.13 (52.88–136.97)	20	64.5	−1.512	0.261
16PD	Left	118.41 (67.78–161.44)	20	131	0.971	0.442
	Right	93.66 (63.28–146.53)	20	97	−0.299	0.765
24PD	Left	103.22 (60.47–137.53)	20	70	−1.307	0.306
	Right	93.94 (72.00–126.28)	20	103	0.322	0.765
32PD	Left	101.53 (77.91–140.34)	20	100	−0.187	0.852
	Right	92.53 (63.84–126.28)	20	108	0.523	0.765
40PD	Left	107.72 (75.94–144.28)	20	–	–	–
	Right	90.00 (66.66–150.47)	20	–	–	–
Expert	Left	165.94 (154.13–186.05)	5	0	−2.023	0.125
	Right	165.94 (109.27–197.16)	5	2	−1.483	0.188
Automated segmentation (vs. expert)	Left	114.75 (111.76–122.24)	5	0	−2.023	0.086
	Right	113.48 (104.03–140.17)	5	4	−0.944	0.345

False discovery rate (FDR) corrected significance values that are *p* < 0.05 are highlighted in bold. FDR correction was performed across hemispheres and PD levels, with separate corrections performed for the expert vs. 40PD tests, and the auto. Seg. vs. expert tests

**Table 2:** Descriptive statistics (median and interquartile range (IQR)) for the Dice score data (the overlap between the rater masks for each PD level and the expert masks, and between the automated segmentations and the expert masks), and Wilcoxon signed rank test results between each PD level and 40PD (therefore there are no statistics for 40PD), with false discovery rate (FDR) correction, and an additional Wilcoxon signed rank test between the automated segmentation and 40PD (no FDR correction) - for this test the median Dice score across raters for each participant in each hemisphere were used, to match the sample size of the automated segmentations (5 participants x 2 hemispheres, *N* = 10)

PD level	Median Dice score (IQR)	*N*	W	z	Sig. (FDR)
0PD	0.26 (0.15–0.38)	40	52	−4.812	**< 0.001**
1PD	0.25 (0.12–0.37)	40	15	−5.309	**< 0.001**
2PD	0.28 (0.12–0.46)	40	31	−5.094	**< 0.001**
4PD	0.38 (0.25–0.50)	40	146	−3.549	**< 0.001**
8PD	0.36 (0.28–0.58)	40	174	−3.172	**0.002**
16PD	0.44 (0.33–0.52)	40	285	−1.680	0.106
24PD	0.44 (0.32–0.58)	40	259	−2.030	0.057
32PD	0.48 (0.36–0.60)	40	447	0.497	0.619
40PD	0.51 (0.33–0.59)	40	–	–	–
Automated segmentation	0.50 (0.38–0.58)	10	32	0.459	0.647 (no FDR)

Significance values that are *p* < 0.05 are highlighted in bold

**Fig. 3:**
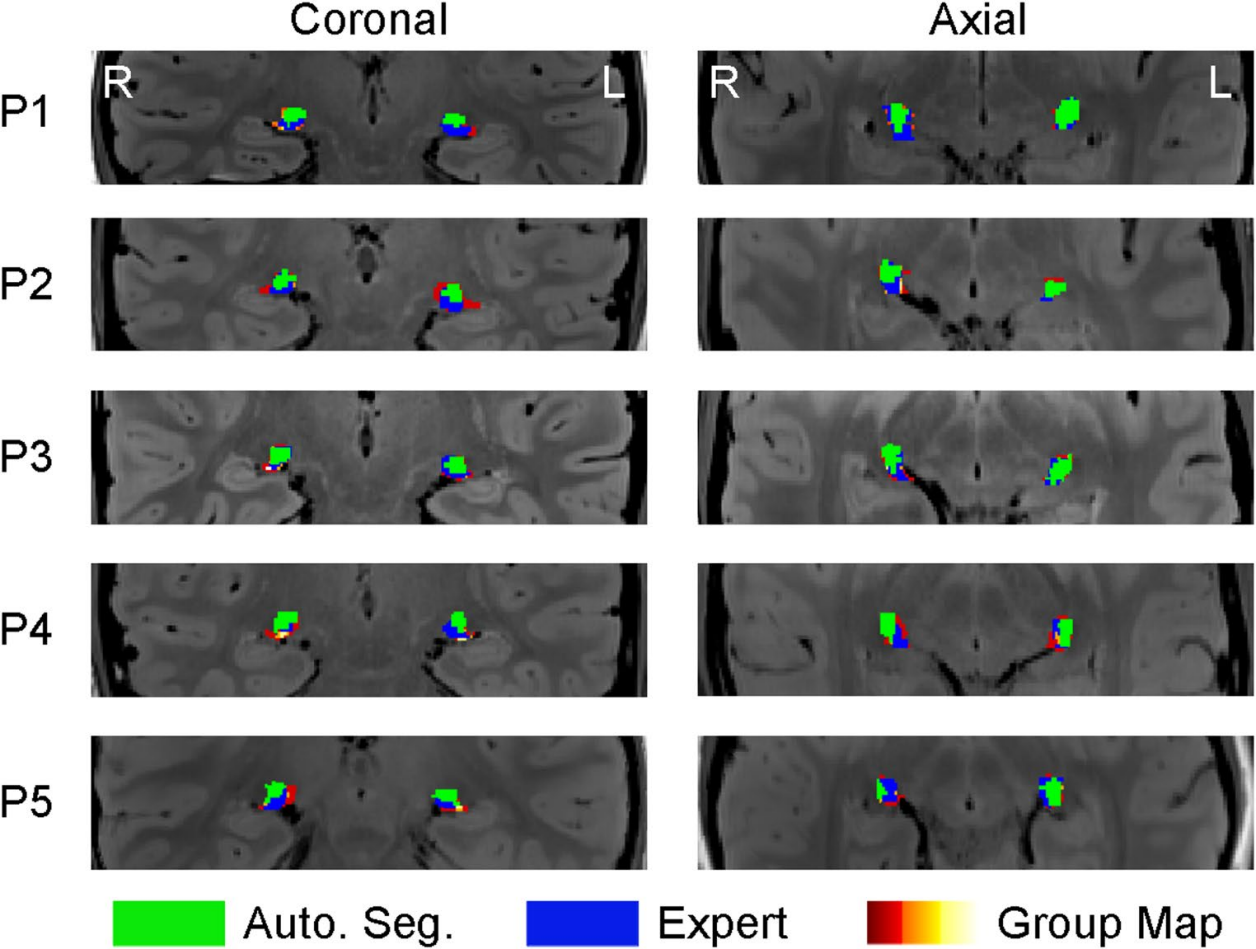
Overlaid LGN masks for one coronal and one axial slice of each participant, which are approximately centred on the LGN (note that the LGN central point differs slightly in each hemisphere). The bottom mask (ranging from red to white based on how many raters included that voxel in their mask, where white is all raters) is the combined raters mask (Group Map), followed by the expert LGN mask (in blue), and the topmost mask is the automated segmentation mask (in green). Participants are numbered P1 to P5 based on the Dice score between the automated segmentations and the expert LGN, with the highest Dice score participant (most overlap between the automated segmentation and expert LGN) numbered P1

The median accuracy (Dice score) of the automated segmentations is almost identical to the manual segmentations, based on the comparison to the expert rater (Fig. 2B(i)). However, on an individual participant level (Fig. 2B(ii)), for three participants the Dice scores were very similar between independent raters and automated segmentation, but for two participants there was a substantial difference in performance, where the automated segmentation outperformed the independent raters for one participant (blue diamond symbol), and underperformed for another (green triangle symbol). For each participant, the overlap in the three LGN masks (combined independent rater masks (group map), the expert mask, and the automated segmentation mask) are illustrated in Fig. 3 for a single coronal and axial slice taken from the approximate centre of the LGNs. From P1 to P5 the participants are ordered from highest to lowest automated segmentation Dice score respectively. These images demonstrate the high overall agreement in LGN location, with some differences in overall spread of the LGN in each hemisphere between the masks.

**Fig. 4:**
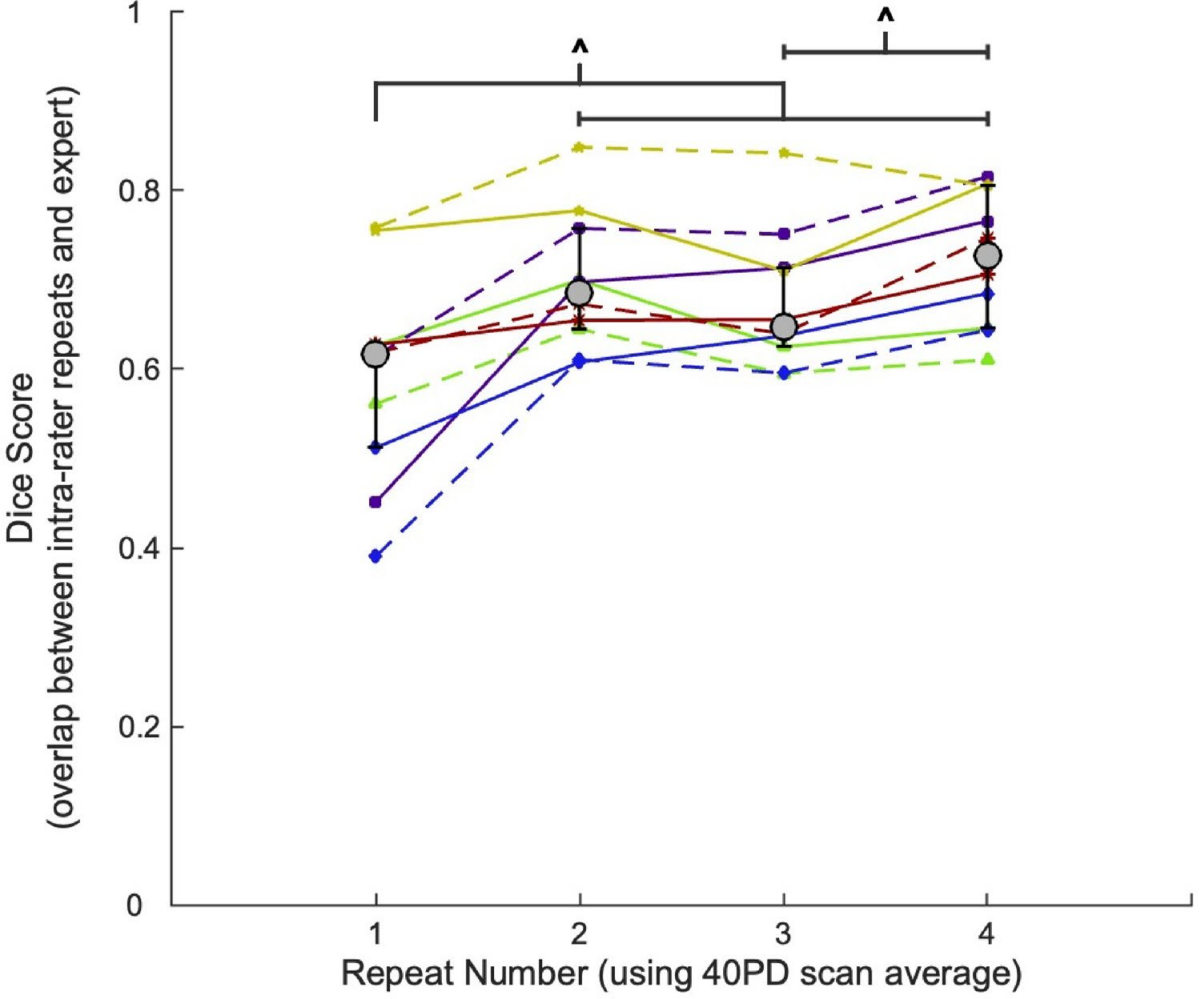
Median Dice score across participants (with interquartile range bars) shown as grey circles with black outlines, for each repetition performed by one rater. Wilcoxon signed rank tests were carried out between each repeat pair combination, and corrected for multiple comparisons using false-discovery rate (FDR) correction. Significant differences between pairs are indicated with ^ (*p* < 0.05). Dice Scores for individual participants for each hemisphere are shown with different colours/symbols (connected with dashed lines for the left hemisphere and solid lines for the right hemisphere) across each repetition

For the intra-rater analysis the same rater repeated the 40PD segmentation for each participant on four different days (Fig. 4) to compare the Dice scores between the repeats (median (interquartile range): repeat 1 = 0.62 (0.51–0.63); repeat 2 = 0.69 (0.64–0.76), repeat 3 = 0.65 (0.63–0.71), repeat 4 = 0.73 (0.65–0.81)). There were significant differences between the first repeat and each of the subsequent repeats, as well as between repeat three and four (Table 3). There were no significant differences between repeats two and three or between repeats two and four, indicating that there is some improvement in reliability with increased repetition of segmentations.

**Table 3:** Wilcoxon signed rank test results from the intra-rater analysis of Dice scores (the overlap between the intra-rater mask repeats and the expert masks) across participants for the 40PD level

	Repeat 1	Repeat 2	Repeat 3	Repeat 4
Repeat 1	–	W = 0 *z* = −2.803 *p* = 0.015	W = 6 *z* = −2.192 *p* = 0.043	W = 0 *z* = −2.803 *p* = 0.015
Repeat 2	–	–	W = 43 *z* = 1.580 *p* = 0.137	W = 13 *z* = −1.478 *p* = 0.139
Repeat 3	–	–	–	W = 3 *z* = −2.497 *p* = 0.025

A Wilcoxon signed rank test was carried out between each possible repeat pair. All *p* values are false discovery rate (FDR) corrected

**Fig. 5:**
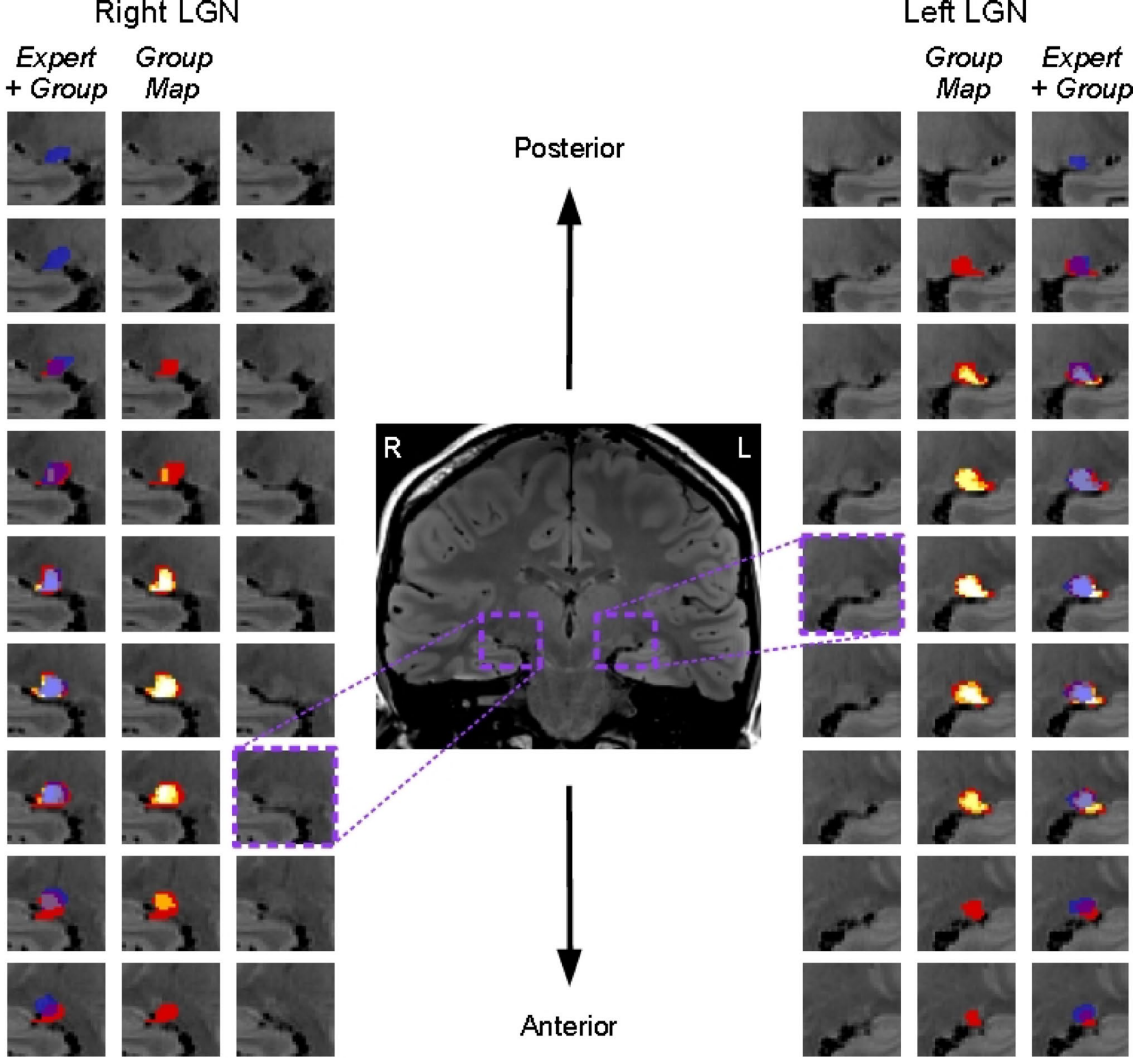
Example rater masks for one participant across all LGN slices in each hemisphere, starting with the most posterior slice at the top (not the same actual slice in each hemisphere). Innermost images are slices from the 40PD scan, for right and left hemispheres (radiological view, so they appear on opposite sides) with no mask overlays. Within each hemisphere, the middle images (labelled ‘*Group Map*’) show the combined rater masks overlaid on the 40PD scan (where colours move between red (1), orange (2), yellow (3), and white (4) based on how many raters included that voxel in their mask), the outermost images (labelled ‘*Expert + Group*’) show the same combined mask as in the middle images, with the addition of the expert rater mask overlaid in translucent dark blue (note that for this participant there are some slices where only the expert selected voxels, for other participants the opposite can also be true where our raters selected voxels in slices where the expert did not select any)

The average Dice scores for the 16PD to 40PD levels ranged between 44% and 51% overlap. To illustrate how the expert masks varied from the rater masks across coronal slices, Fig. 5 shows all the LGN slices in each hemisphere for one participant for the 40PD scan level, with the overlaid group map (combined rater masks), as well as the overlaid expert mask. The white parts of the group map show where all independent raters selected those same voxels as part of their masks; agreement in voxel selection was highest for the central slices. For the representative participant shown, the expert masks tend to extend a shorter distance laterally, but show greater extension in the anterior and posterior directions.

**Fig. 6:**
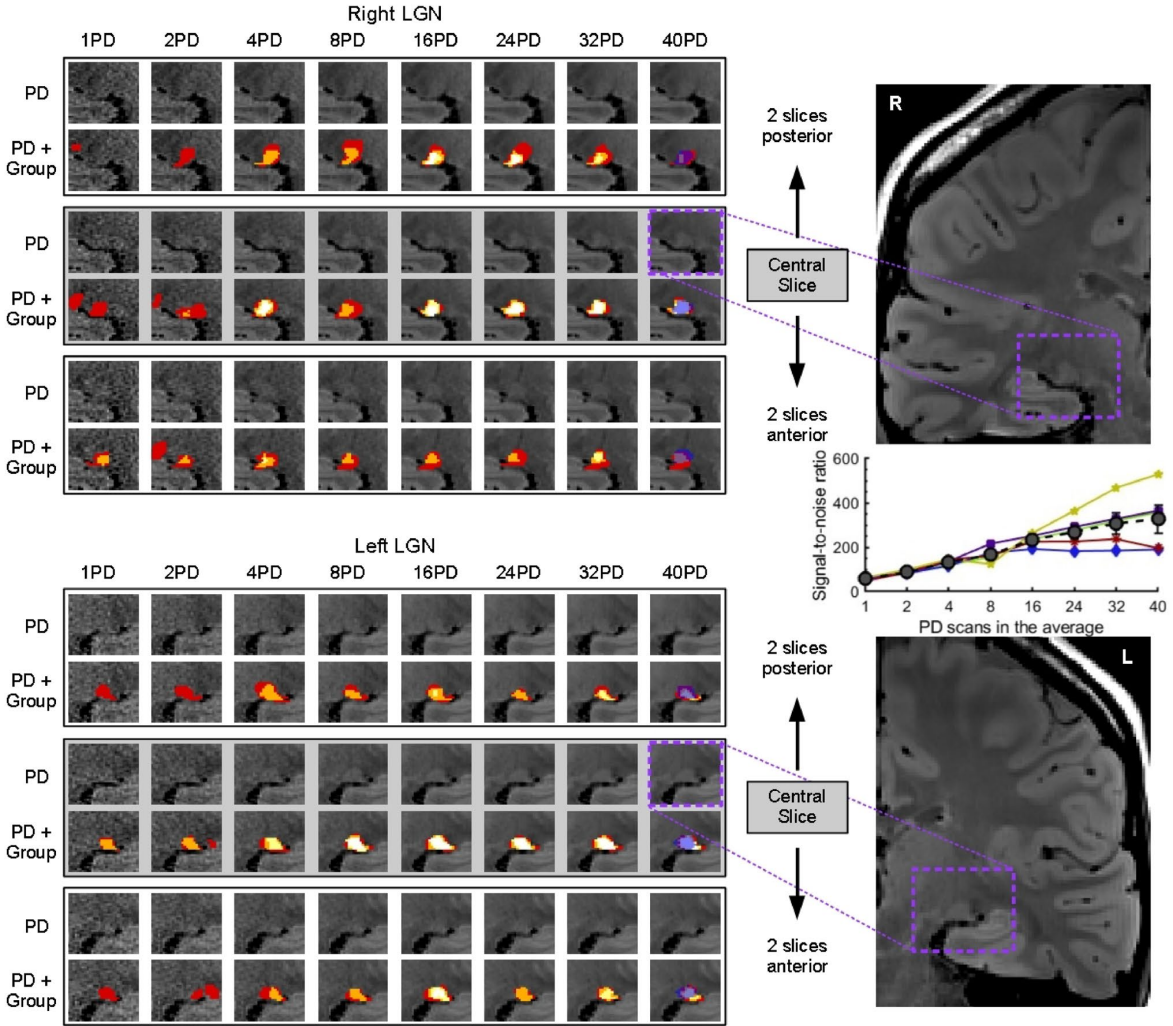
Example of the right and left LGN (top and bottom panels, respectively) across PD scan average levels (number of scans in the PD average) for three example slices - the central slice for this participant’s LGN, and 2 slices away from the central slice in the posterior and anterior directions. For each PD level and slice, the upper image (labelled ‘PD’) shows the average PD without overlays, and the lower image (labelled ‘PD + Group’) shows the same PD image with the combined rater mask (the group map) overlaid (colours move between red (1), orange (2), yellow (3), and white (4) based on how many raters included that voxel in their mask). For the 40PD level the expert mask is also overlaid in translucent dark blue over the combined mask. The rightmost panels show each hemisphere, with a dashed purple box around the LGN area featured in the central slice images. The inset scatter plot on the right, between the hemisphere images, shows the signal-to-noise ratio (SNR) of the average PD image across PD scan levels. Each participant is plotted with a different colour/symbol, with the mean across participants overlaid in dark grey circles with standard error bars (note that for the PD scan levels 1, 2 and 4 the participant data points lie beneath the mean data points, with more participant variation in SNR values for the higher PD levels)

An example of how the image quality (SNR) improves across PD scan levels can be seen in Fig. 6, for the same participant shown previously, on three example slices (the central LGN slice, and slices taken two slices posterior/anterior to the central slice). The image quality noticeably improves with increasing PD scan level, from 1PD to 40PD, and a measure of signal-to-noise ratio can also be seen to increase with more PD scans in the average (see inset scatter plot). Signal-to-noise ratio (SNR) was calculated for each PD scan level in each participant using the expert LGN mask, and an ‘air’ mask created outside of the brain (a combination of two 3D cube masks (5 × 5 × 5 voxels), created in superior lateral locations (left and right) on the coronal slice that was approximately in the centre of the LGN). The mean and standard deviation of the image intensity within each mask, for each PD scan level, was calculated using *fslstats* tools, and the SNR was calculated using the following procedure (Dietrich et al. 2007; Mcketton et al. 2015): SNR = 0.655*µ_LGNtissue_/*σ*_air_. When comparing the masks drawn across PD scan levels, there is a notable difference in the group maps for the 1PD and 2PD scan levels, where at least one of the independent raters draws outside of the LGN locus, and generally smaller masks are drawn.

## Discussion

We demonstrate that the accuracy of LGN masks drawn by independent raters does not continue to improve indefinitely with increasing numbers of PD scans used in the image average. The mask accuracy, as determined by the overlap with an expert mask, is not significantly different between 16PD and 40PD scans in the average.

There were significant differences between the intra-rater segmentation repetitions, notably between the first repetition and all subsequent repetitions, as well as a difference between the third and fourth repetitions. Overall, Dice scores improved with repeated segmentations. It may therefore be advantageous to repeat manual segmentations at least once so that only overlapping voxels (i.e. those voxels that are always included in the mask for each repeat) are used in a final region of interest (ROI).

The automated segmentation benefits from only requiring the T1 structural scan to be performed, and as such is the most time and cost efficient method. However, given that the average accuracy is almost identical to the manual segmentations but with some substantial differences in accuracy on an individual participant level, we propose that manual segmentations are currently still the gold-standard for reliably segmenting the LGN. A minimum of 16 PD scans will produce as accurate an LGN segmentation as 40 PD scans, saving a minimum of 35 min of scan time and there is only a slight additional decrease in accuracy if the total is reduced to 8 scans. The median accuracy (Dice score) is relatively low for masks drawn using just the T1 image (0PD) (0.26, IQR: 0.15–0.38) and those drawn using 1PD (0.25, IQR: 0.12–0.37) or 2PD (0.28, IQR: 0.12–0.46)) images, with the first more notable rise in accuracy occurring from 4PD (0.38, IQR: 0.25–0.50). As such, fewer than four PD scans would not yield any substantial benefit over using a T1 image alone, and may be considered the absolute minimum to produce meaningful LGN segmentations. However, we maintain that our analysis supports a recommendation of 8–16 PD scans for improved accuracy, with no additional benefit from collecting any subsequent scans. We also note that while the Dice scores reach a median of 0.51 (IQR: 0.33–0.59) at the 40PD level, which may be considered a somewhat modest score, with two 3D masks a shift of only one voxel in any direction can have a substantial and disproportionate effect on the Dice coefficient, and so values in the range we report can still indicate good spatial agreement. The most pertinent aspect of the Dice scores in the context of this analysis is how they change with increasing PD level (scans in the average), rather than the absolute value itself.

Finally, our PD data were acquired at an inplane resolution of 0.75 × 0.75 mm x 1 mm. Making the voxels isotropic (1 × 1 × 1 mm) would reduce the thermal noise in each voxel by a factor of approximately 2 while also reducing the overall scan time. This noise reduction is equivalent, in principle, to roughly a factor of 4 increase in the number of volume acquisitions because SNR scales as the square root of the number of volumes. Although the slightly lower resolution may alter the accuracy of segmentation slightly, our data suggest that close-to-optimal segmentation may be achievable with as few as 4 isotropic PD scans.

## Data Availability

The data that support the findings of this study are available from the authors upon request. The data is held securely at York Neuroimaging Centre, University of York, UK.
